# Thermal priming mitigates the effects of lethal marine heatwaves on the Manila clam *Ruditapes philippinarum*

**DOI:** 10.1016/j.isci.2025.113108

**Published:** 2025-07-16

**Authors:** Carmen Federica Tucci, Massimo Milan, Giulia Dalla Rovere, Ilaria Bernardini, Serena Ferraresso, Rafaella Franch, Massimiliano Babbucci, Giovanna Monticelli, Mattia Panin, Tomaso Patarnello, Luca Bargelloni, Luca Peruzza

**Affiliations:** 1 Department of Comparative Biomedicine and Food Science, University of Padova, Viale dell’università 16, Legnaro, 35020 Padova, Italy; 2 Stazione Idrobiologica Umberto D’Ancona, Department of Biology, University of Padova, Padova, Italy; 3 NFBC, National Future Biodiversity Center, Palermo, Italy

**Keywords:** environmental science, ecology, zoology

## Abstract

Climate extreme events like heatwaves (HWs) increasingly threaten marine ecosystems. Using the Manila clam *Ruditapes philippinarum* as a model organism, this study assessed the effects and costs of thermal priming in a realistic scenario. Results showed increased resilience to lethal HWs in primed clams, with significantly higher survival and better defensive behavior compared to naÏve clams. Priming costs were evidenced by a reduced condition index, but hardened animals showed higher antioxidant capacity, upregulation of heat shock response genes, and shifts in microbial composition favoring beneficial taxa. Gene expression analysis revealed environmental memory via upregulation of respiratory chain complex genes, detectable 38 days after priming—the longest reported in molluscs in a controlled setting. Evidence suggests that thermal priming can be an effective mitigation strategy not just for Manila clams but, potentially, for other farmed shellfish species (e.g., oysters and mussels) and for ecological conservation projects against climate extreme events.

## Highlights

• Priming reduced clam death following heatwave that mimicked a realistic event
• Priming had a cost but triggered behavioral and biochemical adjustments in primed clams
• Primed clams showed metabolic upregulation following heatwave and exhibited stress memory
• NaÏve clams had more detrimental bacteria while primed clams had more beneficial taxa

## Graphical abstract

**Figure:**
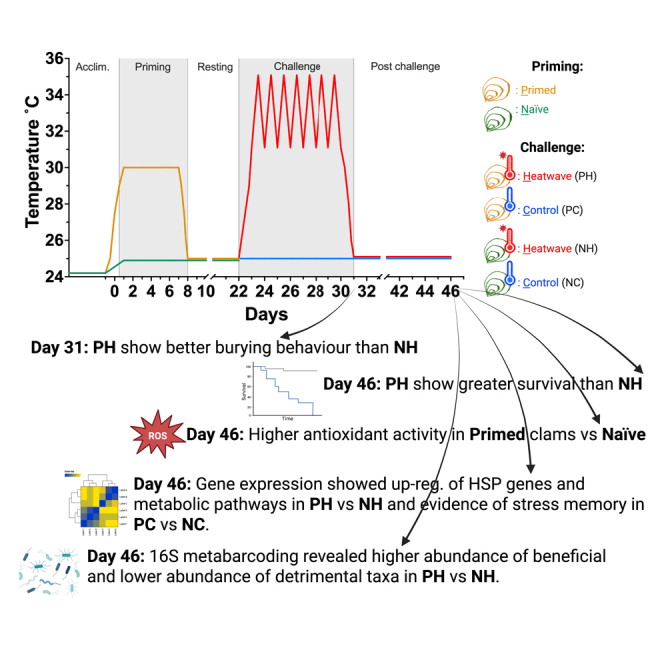

## Introduction

In a world with a fast-growing population, the need to bolster food production is more and more compelling. In this context, bivalve aquaculture provides an excellent source of animal proteins with a very limited carbon footprint.^1^ Bivalves also provide numerous ecosystem services that range from the regulation of water turbidity to nutrient cycling, habitat provision, and carbon sequestration.^2^ Unfortunately, bivalve aquaculture will be heavily impacted by climate change (CC) as it is predicted that, by 2090, coastal areas suitable for this activity will globally decrease by about 10%.^3^ In addition to habitat reduction, ocean acidification and higher prevalence of pathogens are expected to slowly increase in parallel with ramping average temperatures. While such phenomena will certainly have a negative impact on shellfish, CC-related extreme events such as marine heatwaves (MHWs) have rapidly emerged as a much more immediate and dramatic threat to sedentary and sessile species as demonstrated, for instance, by the mass mortality of benthic communities registered in 2003 in the North Adriatic.^4^^,^^5^^,^^6^ Frequency and intensity of MHWs will further increase in the future, multiplying the risks for the benthic fauna.^7^

A possible strategy for mitigating the negative effects of MHWs is constituted by heat-hardening or priming, defined by Bowler^8^ as a transient “adaptation” to a lethal thermal stress following prior exposure to a sub-lethal temperature. Hardening has been extensively studied in plants and other species without a nervous system^9^ as well as in other metazoans (e.g.,^10^^,^^11^^,^^12^^,^^13^) and it represents a form of stress memory that prepares (i.e., “primes”) an organism to future stress by improving its response to adverse environmental conditions. Hardening is a form of phenotypic plasticity as it does not entail any change in the organism genotype and it constitutes a fast “adaptation” response against environmental challenges.^14^ While priming improves the response to future stress conditions, it is generally expected to be associated with some costs^13^; in fact, maintaining stress memory must require changes in regulatory networks to ensure relevant biological systems to be in a “vigilant” state, ready to cope with stressful conditions.^9^ While hardening may represent a general phenomenon naturally occurring in living organisms to better cope with recurrent environmental stress, the physiological and molecular mechanisms of heat hardening are not well understood^15^ and, most importantly, its efficacy in granting higher resilience against MHW that resemble (in terms of daily thermal profile, intensity, and duration) those that occur in nature is, so far, still limited to a handful studies.^11^^,^^12^^,^^16^

Hitherto, great efforts have been devoted toward the understanding of the response to MHWs in marine animals. A plethora of adverse consequences across different animal taxa (e.g.,^17^^,^^18^) beyond mass mortality has been reported, ranging from altered ecosystem services^19^^,^^20^ to impaired reproduction.^21^ In addition, several authors have reported substantial changes in the microbial communities living in several species that generally lead to dysbiosis.^22^^,^^23^^,^^24^^,^^25^^,^^26^ In many studies, MHWs are simulated as acute thermal stress in a high-temperature challenge of relatively short duration that has limited resemblance with environmental conditions in the field (e.g.,^11^^,^^27^). However, as demonstrated by Isotalo et al.^28^ and He et al.,^29^ the consequences of MHWs are largely dependent on their duration and intensity. Hence, it is essential that experiments simulating extreme temperatures closely mirror the temporal and thermal profile of current or predicted MHWs as they occur in the natural environment, in order to draw accurate conclusions on their negative impacts and to test mitigation strategies.

In this framework, the present paper aimed at filling those gaps by exploring the efficacy of heat-hardening in conferring greater resilience against simulated MHWs that mimicked, in terms of intensity and duration, a natural event occurred in 2015 in the Venice lagoon. As model organism, we employed the Manila clam *Ruditapes philippinarum* because of its key role in the ecology of transitional habitats (e.g., lagoons, river deltas) worldwide and its equal importance as highly sustainable food source. Four million metric tons of Manila clam per year are farmed from coastal lagoons and deltas worldwide, supporting a relevant economic sector. Unfortunately, this benthic species is severely threatened by extreme events such as MHW, which are particularly intense in coastal habitats where the majority of clam farming takes place.

To test our hypothesis, we employed an experimental design with four groups: naÏve and primed animals triggered with potentially lethal thermal conditions and naÏve and primed ones without triggering. The priming stimulus was represented by a seven days period at 30°C, a thermal condition that is frequently recorded during summer in the environment where the species is usually grown or found (i.e., lagoons and river deltas). The triggering stimulus closely simulated a real MHW event that occurred in 2015 in the Venice lagoon (one of the major farming sites for this species) with temperatures rising during the day to a maximum of 34°C and then decreasing gradually during the night to a minimum of 31°C. By using an integrative approach, we assessed the effects of priming on fitness-related traits (i.e., behavior, oxidative stress damage, and survival), we measured hardening-associated costs (via changes in condition index), and we elucidated the biological mechanisms underlying priming and stress memory, through the analysis of behavioral, metabolic, biochemical alterations induced by hardening as well as of changes at the transcriptome and microbiome level in the digestive gland, an organ that was reported to be highly relevant in the response of Manila clam to thermal stress.^26^

## Results

### Behavior

**Figure 1:**
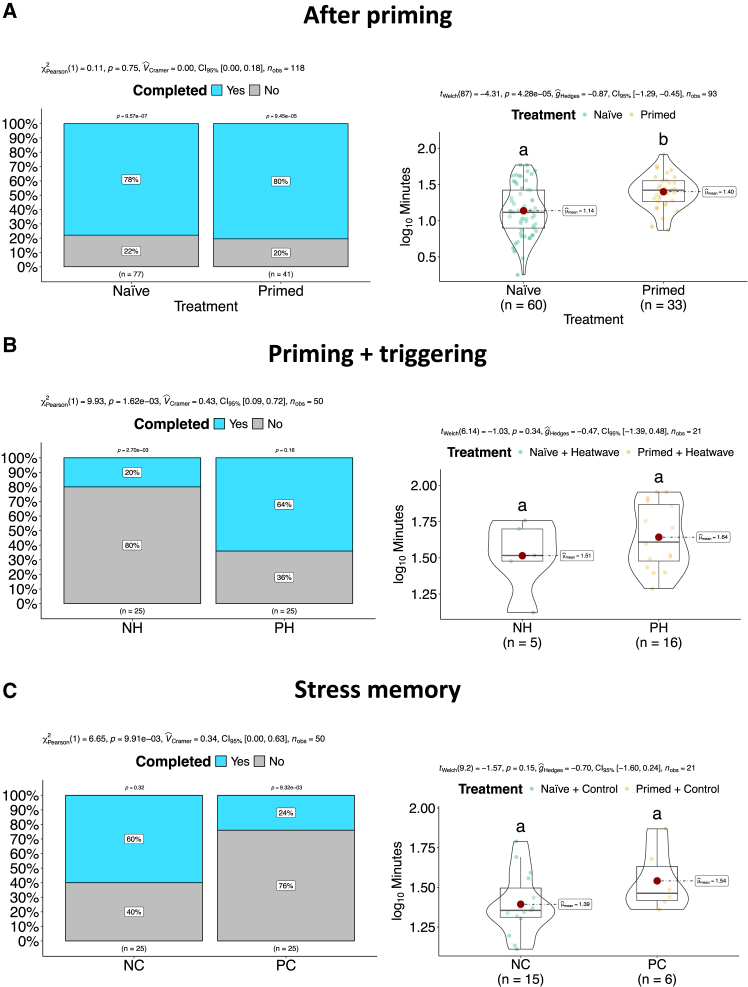
Impact of priming on clam behavior The left panels show the proportion of primed and naÏve clams that successfully buried in the sand (A) after priming and (B) after HW challenge and (C) without HW challenge. The right panels show the burying speed (i.e., the amount of time that individual clams took to hide in the sand) of primed and naÏve clams (A) after priming and (B) after HW challenge and (C) without HW challenge. In left panels (A), (B), and (C) Pearson’s chi-squared test with significance, confidence interval (CI_95%_) and number of observations is reported in the subtitle. Results of one-sample proportion test are displayed on top of each bar. In right panels (A), (B), and (C) box and violin plots with individual observations are depicted. Student’s t test with significance, CI_95%_, and number of observations is reported in the subtitle. Statistical tests are reported in the caption. Different letters indicate significant differences between groups after post-hoc multiple comparisons (significance level: adjusted *p* value <0.05).

Because of its non-invasive nature, the burrowing behavioral test could be applied before the end of the experiment, which was not the case for all the other read-outs. The first test was performed immediately after priming and showed that a similarly high percentage of animals completed the burrowing both in the primed and naÏve clams, although the burrowing speed was significantly slower in primed individuals (Figure 1A). A second round of tests was carried out at day 32 since the beginning of the experiment. When comparing primed and naÏve animals maintained constantly at 25°C, a significantly different behavior was again observed. In this case a lower percentage of primed clams (PCs) had completed the burrowing, while at a similar speed (Figure 1C). The pattern was completely reversed in the comparison between primed and naÏve clams that had experienced the MHW triggering event (Figure 1B). As before, there was no difference in the burrowing speed, but the percentage of animals having completed the process was much lower in naÏve clams after the simulated MHW. Notably, the percentage of HW-triggered primed clams that buried was similar to the percentage of naÏve animals not exposed to the triggering event (Figures 1B and 1C).

### Survival, condition index, and oxidative stress biomarkers

**Figure 2:**
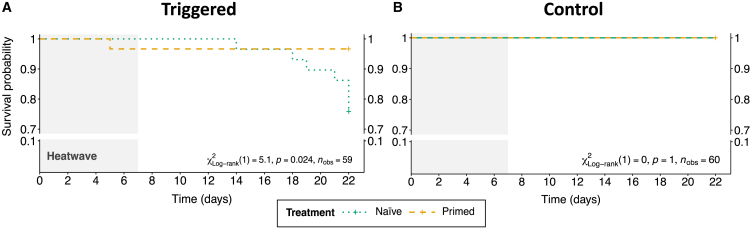
Impact of priming on clam’s survival following MHW conditions Kaplan-Meier survival estimates on primed (yellow dashed line) and naÏve (green dotted line) clams exposed to (A) MHW (gray area) and then kept at 25°C or (B) kept at control conditions (25°C) for the entire time. Differences in survival were computed using Log rank (Mantel-Cox) test. Statistical details (i.e., significance, degree of freedom and number of observations) are reported in the plot. The gray shaded area in (A) indicates the onset and duration of the MHW (refer to Figure S1 for details on MHW conditions). Note that the start time (i.e., day 0) on the *x* axis of both graphs corresponds to day 23 in Figure S1A.

Survival was surveyed for the entire duration of the experiment, including a 15-day post-thermal challenge recovery period at 25°C, since in preliminary trials carried out to optimize experimental settings, it was observed that mortality often occurred after the end of HW challenge. In fact, in naÏve clams exposed to HW significant mortality started one week into the recovery period, while it was negligible in primed animals (Figure 2A) and by the end of the monitoring period, a higher significant mortality rate (∼25%) was found in naÏve animals in comparison to primed ones (∼4%). Control groups showed no mortality at all (Figure 2B).

**Figure 3:**
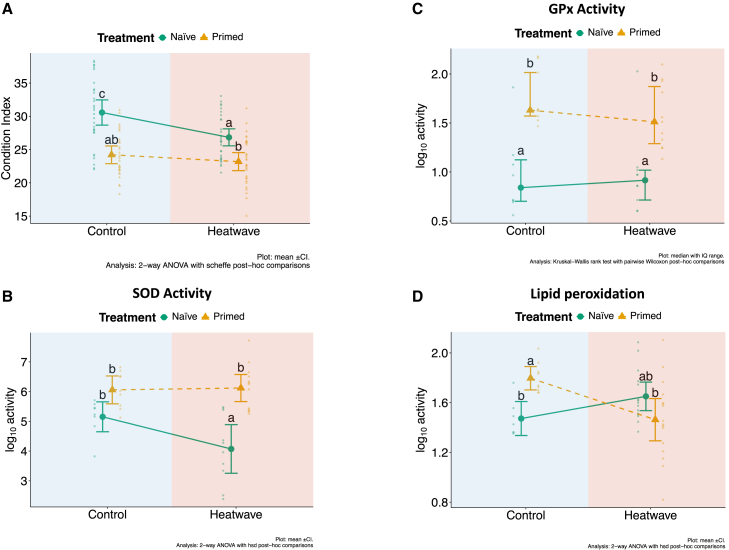
Impact of priming on clam’s overall status and antioxidant defenses (A) Condition index of primed and naÏve clams after challenge. (B–D) Enzymatic activity of SOD (B), GPx (C), and lipid peroxidation (D) in primed and naÏve clams after challenge. For each plot, individual observations are plotted and graphical details (i.e., mean ± CI_95%_ or median ± IQ range) and statistical details are reported in the caption. Different letters indicate significant differences between groups after post-hoc multiple comparisons (significance level: adjusted *p* value <0.05).

Condition index (CI), which indicates the overall physiological status of clams, showed that priming entails a cost, as primed clams showed a significantly lower CI both with and without triggering event (Figure 3A), although the difference was significantly smaller after experiencing HW. In fact, the overall pattern showed that CI dramatically decreased in naÏve clams after being exposed to thermal stress, while it did not decrease significantly in primed animals after the triggering event.

Concerning oxidative stress response, superoxide dismutase (SOD) activity was higher albeit not significantly in primed clams in the absence of HW, while the difference became significant with a decrease in the activity of SOD in naÏve clams exposed to HW (NH) compared to naÏve controls (Figure 3B). The activity of GPx was significantly higher in both primed groups (i.e., PC and PH) in contrast with both naÏve groups (i.e., NC and NH), suggesting that priming induces a stable and trigger-independent expression of this antioxidant enzyme (Figure 3C). Cellular damage as a consequence of oxidative stress was examined in the four groups by assessing the extent of lipid peroxidation. Comparison of primed clams with naÏve ones under non-triggering conditions confirmed that priming comes at a cost, as a significantly higher amount of peroxidation in primed versus naÏve clams was observed (Figure 3D). In naÏve animals the triggering event had a limited effect, with a non-significant increase in lipid peroxidation between primed and naÏve animals. When comparing lipid peroxidation between the two primed groups, animals that were exposed to the triggering stress paradoxically showed a significant decrease in comparison to non-triggered animals.

### Transcriptome analysis

**Figure 4:**
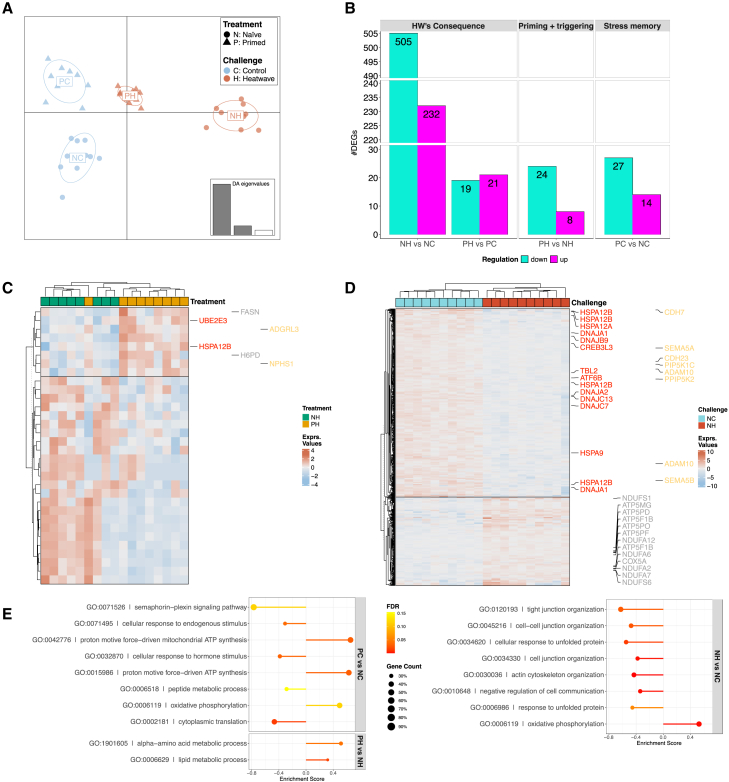
Impact of priming on clam’s gene expression (A) Discriminant analysis of principal components (DAPC) showing the distribution, along the first two component of variation, of each biological sample (individual points). Points are shape-coded according to their treatment (circles: naÏve; triangles: primed) and are color-coded according to the challenge (red: exposure to MHW; blue: no MHW). (B) Numbers of differentially expressed genes of selected pairwise comparisons, split by up-regulated (purple) and down-regulated (light blue) genes. (C and D) Heatmaps of differentially expressed genes in the “PH vs. NH” (C) and “NH vs. NC” (D) comparisons. Gene names on the right side of the heatmap are added to label genes involved with molecular chaperones (red), metabolism (gray) and cell-junction (yellow). Annotation on top of the heatmap illustrates the membership of individual samples to each treatment. (E) Functional analysis (i.e., GSEA) associated with the different pairwise comparisons (reported in the gray, right column). A positive enrichment score indicates up-regulation, while a negative enrichment score indicates down-regulation. Significant hits are color coded according to their FDR value and their size is proportional to the percentage of genes contributing to the observed enrichment score for the pathway/process.

Transcriptional profiles were analyzed in the four experimental groups at the end of the final recovery phase, depicting the medium-term effects on gene expression of priming and/or the triggering event. The digestive gland was analyzed here as previous studies showed that this organ is significantly involved in the clam response to HWs. A discriminant analysis of principal components (DAPCs) was used to explore overall patterns, with a clear separation of samples into four clusters, overlapping the experimental groups (Figure 4A). Evidence from the DAPC suggested that naÏve animals experiencing the triggering event showed the most relevant transcriptional changes. Such evidence was confirmed when significant differentially expressed genes (DEGs) were identified in all possible pairwise comparisons, as the number of DEGs was substantially higher when examining the effects of HW in naÏve clams (Figure 4B). Full lists of DEGs for each comparison are reported in Tables S1A–S1D.

The comparison between PC and NC groups revealed genes and gene networks potentially involved in thermal stress memory as several significant DEGs and gene sets were observed over a month (38 days) after the end of the priming event. Noteworthy, among DEGs were two chaperones, *HSPA12A* (fold change (FC) 4.5, false discovery rate [FDR]<0.01) and *TRAP1* (FC 0.4, FDR<0.05). The former belongs to the hsp70 family, the latter to the hsp90 one. Remarkably, there was no overlap between DEGs identified in the comparison between primed and naÏve clams after the triggering event and those differentially regulated just by the priming. As external and internal stimuli often affect biological processes through modest, but coordinated expression changes in the set of genes that underlie those processes, a gene set enrichment analysis (GSEA) was also implemented here (Tables S1E–S1H). Gene network analysis via GSEA showed different sets of genes that were significantly regulated. Negative regulation of protein translation (GO:0002181 *cytoplasmic translation*, normalized enrichment score (NES) −1.9, FDR = 0.01) and positive regulation of ATP synthesis (GO:0015986 *proton motive force-driven ATP synthesis*, NES 2.1, FDR = 0.03; GO:0042776 *proton motive force-driven mitochondrial ATP synthesis* NES 2.2, FDR = 0.03) were observed. Two larger and partially overlapping gene networks underlying cell response to stimuli (*GO:0032870 cellular response to hormone stimulus*, NES -1.7, FDR = 0.03; GO:0071495 *cellular response to endogenous stimulus*, NES -1.5, FDR = 0.04) appeared both to be down-regulated (Figure 4).

In the comparison between primed and naÏve animals that both experienced the triggering event (i.e., PH and NH), several genes were differentially regulated (Figure 4B) over two weeks after the HW challenge ended, including the up-regulation of genes potentially implicated in thermal tolerance (Figure 4C): *HSPA12B* (FC 1.8, FDR = 0.04), a heat shock protein (HSP); *NPHS1* (FC 2.1, FDR = 0.01), a molecule involved in the regulation of epithelial barriers; *ADGRL3* (FC 3.5, FDR = 0.04), which plays a role in cell-cell adhesion; *FASN* (FC 9.8, FDR = 0.02), which is involved in fatty acid synthesis and endothelial permeability. Broad gene sets were found to be significantly enriched at FDR<0.05 (Figure 4E), involving lipid and amino acid metabolism (GO:0006629 *lipid metabolic process*, NES 1.5, FDR = 0.02; GO:1901605 *alpha-amino acid metabolic process*, NES 3.8, FDR = 0.04). Both gene networks were up-regulated.

In the comparison between the two naÏve groups (i.e., NH and NC), we found many down-regulated genes in NH linked to heat shock response (e.g., *HSPA12B*, *HSPA12A*, *HSPA9*, and *DNAJB9*) and to epithelial barrier permeability (e.g., *SEMA5B*, *SEMA5A*, Figure 4D). In NH, GSEA highlighted a significant down-regulation of pathways associated to protein folding and protection (GO:0034620 *cellular response to unfolded protein*, NES -1.9, FDR = 0.01; GO:0006986 *response to unfolded protein*, NES -1.7, FDR = 0.05, Figure 4E) and to pathways linked with cellular adhesion and structure (GO:0030036 *actin cytoskeleton organization*, NES -1.9, FDR <0.0001; GO:0034330 *cell junction organization*, NES -1.7, FDR = 0.001; GO:0120193 *tight junction organization*, NES -1.8, FDR = 0.03), such as “cell-cell junction organization”, “tight junction organization”, “actin cytoskeleton organization”, and “cellular response to unfolded protein” (Figure 4E).

### Clam-associated microbiota

**Figure 5:**
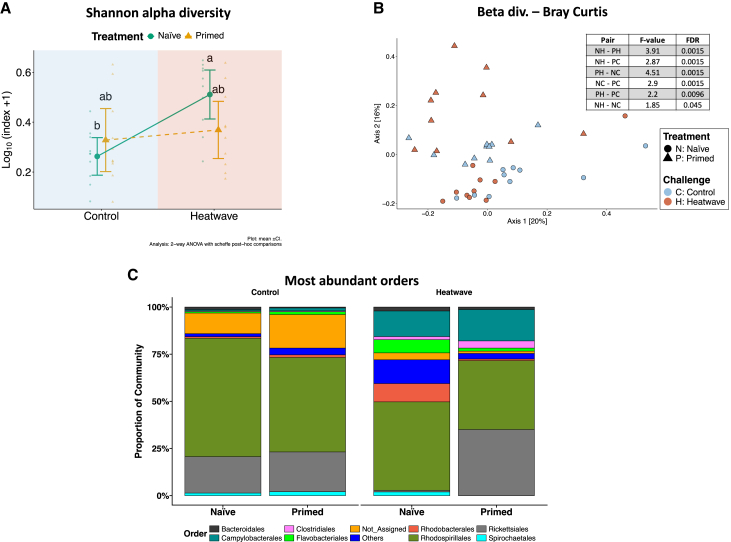
Impact of priming on clam’s microbiota (A and B) Effects on alpha (A) and beta (B) diversity of primed (triangles) and naÏve (circles) clams. (C) Taxonomic composition of the average microbiome abundance at order level. Bar plot of significant genera (genera with relative abundance <5% are not shown). In (A) mean ± CI_95%_ and individual points are plotted. Statistical details are reported in the caption. Different letters indicate significant differences between groups after post-hoc multiple comparisons. In (B) points are shape-coded according to their treatment (circles: naÏve; triangles: primed) and are color-coded according to the challenge (red: exposure to MHW; blue: no MHW).

The microbiota associated with the clam digestive gland was characterized in all four experimental groups. Shannon alpha diversity showed a significant increase in naÏve clams after exposure to HW (Tables S2A–S2D, Figure 5A). The beta diversity was calculated using the Bray-Curtis index at the genus level and visualized with a PCoA (Figure 5B). NaÏve and primed clams appeared to be separated along the second axis, irrespective to the presence or absence of the triggering event, although PERMANOVA revealed significant differences in all pairwise comparisons (Figure 5B insert).

Analysis of taxonomic composition showed that the microbial community of NH animals was characterized by a conspicuous relative abundance of bacteria of the orders *Flavobacteriales* and *Rhodobacterales* (Figure 5C), while *Clostridiales* were highly abundant in PH clams. At the genus level, pairwise comparisons by means of multiple linear regression with covariate adjustment between PH and NH highlighted the presence of 32 differentially abundant genera. Several of these genera, which are included in the putatively detrimental orders of the *Flavobacteriales* and *Rhodobacterales*, were indeed more abundant in naÏve clams after the triggering event (Table S2A), while several taxa belonging to the potentially beneficial order *Clostridiales* were more abundant in primed and triggered clams. The comparison between PC and NC showed long lasting effects of priming on clam-associated microbiome, with two over-represented taxa in PCs and one more abundant in NC.

## Discussion

Nowadays it is widely recognized that extreme climate events such as MHWs will strike at increasing frequency, duration, and intensity worldwide,^19^^,^^30^ with great impact on marine biota. In this context, priming and environmental stress memory represent a biological mechanism to potentially cope with such extreme events. Priming is known to the scientific community since the ‘70s, although the majority of research in multicellular organisms has mostly focused on plants.^9^ In metazoans, possibly the best studied case is that of corals.^31^ In this taxonomic group, it emerged that priming and environmental stress memory might represent a more rapid strategy than genetic adaptation for sedentary and sessile animals to counteract recurrent stressful episodes without relocation. It could be envisaged as an operational tool to obtain more resilient animals before they are seeded in the natural environment either in the context of restoration programmes or for aquaculture purposes.

Analysis of thermal priming in corals also highlighted the potential importance of the host-associated microbial communities in stress memory,^32^ although the relevance and cause-effect links remain to be fully elucidated. Studies on more complex marine animals are unfortunately still scarce and the available ones often follow an experimental design where short (i.e., hours) sub-lethal exposure times at fairly high temperatures are used to induce priming. Likewise, triggering stimuli generally consist of acute heat shock events often at non-realistic temperatures rather than conditions resembling those in a natural HW event (e.g.,^27^^,^^33^^,^^34^^,^^35^). This approach limits the understanding of the potential of priming in counteracting natural extreme events both as an operational tool and a natural mechanism of acclimatization. In this work, as already mentioned, priming conditions were implemented resembling in length and intensity the average sublethal MHWs currently recorded in transitional zones along the coast of the Adriatic Sea and, in parallel, the triggering stimulus closely simulated a lethal MHW that occurred in 2015 in the same area.

All three fitness-related read-outs measured in this work (i.e., behavior, mortality, and oxidative stress) showed a clear beneficial effect of priming after the triggering stimulus. As reported by Luo et al.,^36^ “behavior is the most direct response of an organism and reflects the prospect of surviving” and, in clams, is particularly relevant as it allows them to evade predators and helps mitigating the effects of high-water temperatures. Recent studies showed behavioral responses of naÏve animals were significantly impaired following MHW exposure.^26^^,^^35^^,^^36^^,^^37^^,^^38^ The potential fitness costs of priming are clearly appreciated as burrowing speed slowed down immediately after priming, while in the longer term it drastically reduced the percentage of burrowed clams (Figures 1A and 1C) compared to untriggered naÏve animals. However, as already noted, the pattern was entirely reversed in the presence of the triggering event, with the percentage of completed burrowing events significantly higher in primed clams (a percentage that closely mirrored that recorded in untriggered naÏve animals, Figure 1B). Survival, the trait most directly affecting animal fitness, was significantly greater in comparison to naÏve clams. Mortality was observed only after the triggering event, therefore there seemed to be no priming-associated costs in terms of survival (Figure 2). The third fitness-related readout, oxidative stress damage measured through lipid peroxidation, showed a pattern similar to that recorded for burrowing behavior: at the end of the experiment, hardened clams displayed a significantly higher cell membrane damage in the absence of a triggering event. However, hardened and triggered animals had a lower level of damage, comparable to naÏve ones (Figure 3D).

The fact that priming entails biological costs, as expected based on existing literature,^9^ was clearly evident from the analysis of condition index, which was significantly lower in primed clams compared to naÏve ones when priming was not followed by a triggering stimulus (Figure 3A). The ratio between soft body weight and shell weight, the condition index (CI) used here, is an indicator of energy storage in bivalves.^39^ Lower CI in primed clams suggested higher energetic demand, perfectly fitting the energy budget hypothesis, where changes necessary to withstand harsher environmental conditions require an energy investment of the organism, which reallocates energies from other processes.^40^^,^^41^ However, it is interesting to note that this energetic cost was not paid again when primed animals were exposed to the triggering stimulus, as there was no difference in CI between PH and PC (Figure 3A). The metabolic costs of priming and stress memory maintenance paid off as a potentially lethal HW appeared to entail no extra energetic cost. On the other hand, naÏve clams spent a significant amount of energy after exposure to the triggering event even if, such energetic costs, were not sufficient to prevent a significantly higher mortality in these animals. The other fitness-related measures, burrowing behavior and oxidative stress damage, showed a similar, even more pronounced pattern in naÏve clams.

The energetic investment was likely utilized by primed animals to face both the priming stimulus, which is in itself a stressful condition that induces a response in the animal, and to set up the regulatory changes to maintain stress memory. The comparison at the molecular and enzymatic level between naÏve and primed clams in the absence of triggering stimulus more than a month after priming revealed at least part of such changes. Enzymatic activity of SOD was higher, but not significant, in primed animals without triggering stimulus, although SOD activity significantly decreased in naÏve clams as a long-term consequence of HW exposure, while it was unaffected in primed ones. The activity of GPx was significantly higher in primed clams irrespective to the occurrence of HW (Figure 3). Thermal stress is known to increase reactive oxygen species (ROS), which are detrimental for cells because they cause oxidative damage to membranes and organelles.^42^ In fact, many authors have reported an increased activity in the components of the antioxidant pathway (e.g., SOD, GPx, and Cat) in the acute response to MHWs (e.g.,^15^^,^^26^^,^^43^^,^^44^^,^^45^). Primed clams appeared therefore to be better equipped for oxidative stress response than naÏve ones, with priming being able to confer higher protection against ROS, as reported by others.^15^^,^^46^ When searching for differential mRNA expression specifically for GPX- and SOD encoding genes, five isoforms of GPX (*GPX1*, *GPX2*, *GPX3*, *GPX5*, and *GPX6*) were found in the annotated clam genome. Only *GPX5* showed marginally significant differential expression with FC = 1.6 (uncorrected *p* = 0.055). Several SOD isoforms were also identified, although none appeared to be differentially regulated, mirroring the results of SOD enzymatic activity.

While antioxidant response did not show a specific profile at the gene expression level, RNA sequencing (RNA-seq) analysis revealed a vast transcriptional program induced by priming, which likely underlies environmental stress memory. At the single gene level, the involvement of one protein chaperone confirmed the importance of maintaining protein quality during thermal stress. The observed negative regulation of cytoplasmic protein translation is fully in agreement with the known phenomenon of general protein synthesis reduction as a response to most types of cellular stress. In general, such phenomenon is mediated by post-translational changes, e.g., through phosphorylation of EIF2a, which ensure a faster response. Stress-induced translation repression represents a protective mechanism to ensure protein quality, while allowing reallocation of cellular resources toward the synthesis of specific stress proteins.^47^ In the case of stress memory, evidence from the present work suggested that the same goal might be achieved on the longer term through the coordinated down-regulation of several genes involved in protein translation, including key translation initiation factors (e.g., *EIF4G*, different components of the EIF2 complex), ribosomal proteins, and proteins with an important regulatory role in translation control such as *RACK1*, a scaffold protein that transduces upstream signals to translation, and mTOR.^48^

Two other broad sets of genes were significantly involved in clam environmental memory. These gene sets are largely overlapping and include several genes encoding protein subunits of respiratory chain complex I and complex V (ATP synthase), which were all up-regulated in primed clams. Expression of genes encoding respiratory complexes subunits have been shown to be regulated by a broad array of non-coding RNAs (i.e., miRNAs, lncRNAs, and ciRNAs^49^), which are considered to play a key role in plant stress memory.^50^ Complexes I and V represent respectively the first and the last component of the respiratory chain, the core of the mitochondrial machinery for energy production in eukaryotic cells. A possible interpretation of this finding is that coordinated upregulation of several components of these two complexes of the electron transport chain might help facing the increased energy demand during heat stress and reduce the associated production of reactive oxygen species that is known to occur under such condition. In murine cardiomyocytes upon anoxia/reoxygenation treatment, which induces oxidative stress like thermal stress, the cellular response is mediated by *miR-762* and leads to decrease complex I activity, lowering intracellular ATP, increasing ROS levels, and promoting apoptotic cell death.^51^

Evidence that repressing cell response might improve stress tolerance has often been reported and has provided the basis for the hypothesis of hormesis. It might also explain the observed down-regulation of two other complex gene sets, which both include a large number of genes that participate in the response to hormonal/internal stimuli. Of note is the repression of *SIRT1* in primed clams. Sirtuin1 belongs to the class III histone deacetylase family of proteins and is crucial for various biochemical processes within cells, including oxidative stress, inflammation, lipid metabolism, and autophagy as recently reviewed by Wang et al.^52^ for hepatic cells, which functionally recapitulate the role of clam hepatopancreas. Since *SIRT1* functions both indirectly through epigenetic modifications of regulatory regions in several target genes and directly through deacetylation of key transcription factors, its effects on transcriptional programs are potentially very broad.

The comparison of primed and naÏve clams after the end of the triggering event also showed a significant divergence in their transcriptome profiles, suggesting that priming and stress memory translate into a modified response to thermal stress, targeting key cell biological processes. As exposed by Moseley,^53^ when the body is not able to deal with the excessive heat, then this stressful condition can cause tissue injury. Some tissues, in particular the gut, must preserve their integrity to fulfill their barrier function between the external and the internal environments. For instance, excessive heat can damage the intestinal epithelial barrier and induce a release of endotoxins in the body.^53^^,^^54^ Animals that are acclimatized to their environment have a greater ability to effectively disperse heat excess, thus avoiding damage to epithelial barriers, mostly thanks to HSPs, which prevent denaturation of proteins and lower barrier permeability.^53^ In PCs exposed to heat stress, the observed up-regulation of *HSPXX* and *NPHS1* and *ADGRL3*, two regulators of barrier permeability suggested a more efficient acclimatory response. Recent studies reported upregulation of the tight junction pathway, cell adhesion molecules, and of several HSP genes in heat-hardened *Mytilus coruscus*,^55^ *Argopecten irradians irradians*,^56^ *Crassostrea gigas*,^57^ and *Pinctada maxima*.^58^ Our hypothesis is further supported by the fact that an opposite response (i.e., the down-regulation of six heat shock proteins and many molecular chaperones and the down-regulation of processes such as “cell junction” and “response to unfolded protein”) was observed in naÏve clams (NH) after the triggering event, reinforcing the idea that these changes (i.e., preventing protein denaturation with HSPs and maintaining epithelial barrier integrity) at molecular level were indeed acclimatory responses that play an important role in dealing with heat damage.

In order to maintain the internal homeostasis and preserve normal functions, the body requires energy to enable stress adaptation,^40^^,^^59^ hence metabolic adjustments are foreseen when animals are exposed to stress. In the present study, up-regulation of two large sets of genes involved respectively in lipid and amino acid metabolism suggested that primed clams exposed to lethal HW might be able to mount more quickly than naÏve animals a metabolic response in the digestive gland, which is fundamental in food digestion and in storing energy-rich compounds, using non-sugar compounds, such as lipid and amino acids, putatively to sustain the production of ATP for the entire body. Recently, Georgoulis et al.^15^ and^60^ showed that priming *Mytilus galloprovincialis* resulted in metabolic adjustments to increase ATP production, by acting on the Krebs cycle and on the electron transport system with responses that were either stronger, earlier, faster or more sensitive than naÏve animals. Furthermore, as reported in Gurr et al.,^10^ clams primed to acidified conditions or thermal stress, respectively, were able to respond more quickly to a second encounter with lethal stress by triggering genes enriched for mitochondrial recycling and immune defenses. In contraposition to priming with sub-lethal thermal stress, He et al.^61^ showed that repeated exposures to extreme thermal stress in the pearl oyster induce a general down-regulation of energy metabolism and argued that this may be linked with a dysfunction of mitochondria. This latter observation highlights the difference between repeated exposure to extreme (and potentially lethal) heat stress and priming via non-lethal stress. In the non-lethal treatment animals can trigger transcriptomic changes and frontload genes for important pathways such as metabolic pathways (e.g.,^10^), while repeated lethal treatments damage to key cellular systems for energy production and regulation of metabolic processes might be too extensive for priming being beneficial. In fact, in corals it has been observed that excessive stress exposure during priming have detrimental effects in the recovery phase and after a triggering event.^31^

As already observed mainly in corals, priming and triggering event induced significant changes in clam-associated microbiota. The role of host-associated microbiota is increasingly recognized as fundamental in animal health and disease.^62^^,^^63^ Despite the digestive gland microbiome shows a remarkable resilience to change,^64^ it was recently proved that sub-lethal, prolonged HWs can induce a substantial shift in its composition, resulting in increased relative abundance of detrimental and concomitant decrease of beneficial taxa.^26^ In this study, taxa belonging to the families *Flavobacteriaceae*, *Cryomorphaceae*, and *Rhodobacteraceae* (Figure 5), identified as detrimental by Vompe et al.^22^ in corals, were more abundant in naÏve clams after exposure to HW, suggesting in line with previous studies,^26^^,^^65^ that HW can induce dysbiosis by favoring the onset of detrimental taxa. As reported by Scanes et al.^25^ on the Sydney rock oyster, the ability of the host in contrasting the onset of pathogenic bacteria and the parallel decrease of beneficial one is an essential aspect to predict the host ability to survive thermal stress. PCs showed no such increase in relative abundance of detrimental taxa after the triggering stimulus, whereas a higher abundance of beneficial bacteria belonging to the order *Clostridiales* was observed. This is in agreement with results from Liu et al.,^65^ who reported that oysters resistant to heat stress were enriched for bacteria of the orders *Clostridiales* and *Verrucomicrobiales*. In the absence of triggering stimulus, significantly higher abundance of *Clostridiales* was detected in primed clams compared to naÏve ones. It should be noted, however, that the presence of putatively beneficial taxa in the digestive gland microbiome of PCs does not prove that the microbial community has a positive causal effect on the host fitness traits. Proving or disproving such effect would require the creation of naÏve gnotobiotic clams, harboring either a “primed” microbiome or a “naÏve” one.

**Figure 6:**
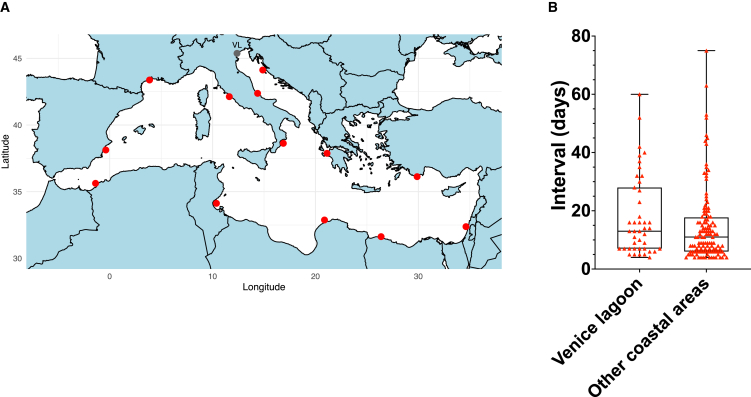
Heatwave characteristics across the Mediterranean basin (A) Map of the Mediterranean basin where 13 random coastal areas (red dots) and the Venice lagoon (gray dot labeled “VL”) have been investigated for the occurrence of MHW over the period June 1^st^ 2000 to June 1^st^ 2024. MHW data for each location were retrieved from https://www.marineheatwaves.org/tracker.html#. (B) Boxplot showing the interval gap (in days) between the end of one MHW and the beginning of the following one for the Venice lagoon and for all coastal areas indicated in (A). Each red triangle represents an interval between two MHW event. Only MHW occurring during summer were considered.

As already mentioned, in the present study the triggering stimulus closely mirrored naturally occurring HWs. The gap of 15 days between priming and triggering recapitulates consecutive MHW events observed in the wild (Figures 6A and 6B). As reported by Moyen et al.,^11^ the majority of MHW occurring in the same year in the Bay of Monterey had a gap interval between consecutive events (in term of days) of less than 16 days. In the Mediterranean Sea, a similar pattern was observed: summer MHWs had a median value for the time gap of 10–12 days (Figure 6B). A similar pattern was found in the Venice lagoon, which together with the Po River delta is the most productive site in Europe for Manila clam. Based on the evidence obtained here it could therefore be hypothesized that “artificial” priming, induced before seeding clams in on-growing areas by artificially raising water temperatures in pre-fattening structures (e.g., flupsies), might represent a highly effective operational tool to mitigate the effects on climate change on clam aquaculture.

### Limitations of the study and conclusion

In this study, it was shown under realistic conditions that priming might have a great potential for mitigating the effects of climate extreme events. In fact, we report increased resilience to a lethal HW that resembled (in terms of intensity and duration) an event occurred in the Venice lagoon in 2015. A complex interplay of adjustments at behavioral, physiological, and molecular levels underpins this increased tolerance, which could be exploited to increase the thermal performance of clams and also to support clam aquaculture. However, in this framework, much remains to be done especially in relation to its optimization under different priming conditions (i.e., intensity, duration). Finally, since clams reproduce during summer when HWs occur, it should be tested whether at the end of the reproductive season, when energy reserves have been depleted by the reproduction, priming costs for maintaining stress memory and ensuring an improved response to triggering stimuli might be excessive for the animal’s energy budget. However, overall priming holds great promise as a mitigation strategy that can be applied also to other important farmed shellfish species (e.g., oysters, mussels) to help sustain the aquaculture sector against climate change induced threats.

## Resource availability

### Lead contact

Further information and requests for resources and reagents should be directed to and will be fulfilled by the lead contact, Luca Peruzza (luca.peruzza@unipd.it).

### Materials availability

This study did not generate new unique reagents.

### Data and code availability

• All next-generation sequencing data have been deposited at the NCBI data repository and are publicly available as of the date of publication. Accession numbers are listed in the key resources table. All remaining data can be shared by the lead contact following request.
• This study did not generate any original code.
• Any additional information required to reanalyze the data reported in this paper is available from the lead contact upon request.

## Acknowledgments

Authors are grateful to Cristina Breggion and Andrea Sambo for technical assistance at the aquarium facility and to Lino Perin for the invaluable assistance with the temperature regulators. This work was supported by the “Seal of Excellence @UNIPD” grant for the project MANILA-SAVE awarded to L.P.; by the “Supporting Talent in ReSearch@University of Padua” grant for the project ASAP (funding from “Ministero dell’Istruzione e della Ricerca”, CUP: C95F21009990001) awarded to L.P.; by the project PRIMECLAMS from “10.13039/100007479Fondazione Cassa di Risparmio di Padova e Rovigo” (CUP: C13C21000160005) awarded to L.B.; further, this work was Co-funded by the 10.13039/501100000780European Union grant IGNITION (GA 101084651) and the UK Research and Innovation (UKRI) awarded to MM. Views and opinions expressed are however those of the authors only and do not necessarily reflect those of the European Union, the Research Executive Agency (REA) or the UKRI. Neither the European Union nor the granting authorities can be held responsible for them. This project was additionally funded under the National Recovery and Resilience Plan (NRRP), Mission 4 Component 2 Investment 1.4 - Call for tender no. 3138 of 16 December 2021, rectified by Decree n.3175 of 18 December 2021 of Italian Ministry of University and Research funded by the European Union – NextGenerationEU.

## Author contributions

L.P. and L.B. conceived the project; C.F.T., M.M., G.D.R., I.B., S.F., R.F., M.B., M.P., and L.P. performed the experiments and collected samples; C.F.T. analyzed behavioral data; C.F.T., R.F., and G.D.R. performed biomarker and molecular analyses; C.F.T. and L.P. performed RNA-seq and 16S microbiota analyses; L.P. wrote the first draft of the manuscript with input from C.F.T., M.M., and L.B.; all authors commented and critically revised the manuscript.

## Declaration of interests

The authors declare no competing interests.

## STAR★Methods

### Key resources table

**Table:** 

REAGENT or RESOURCE	SOURCE	IDENTIFIER
**Critical commercial assays**		

SOD Assay test: Dismutase Activity Assay Kit	Sigma	CS0009
Lipid peroxidation Assay	Sigma	MAK085

**Deposited data**		

RNA-seq and 16S metabarcoding data:	BioProject ID: PRJNA1132140	NCBI - SRA Archive

**Experimental models: Organisms/strains**		

Manila clam. Adult organisms.	Animals bred in a commercial hatchery were used.	SATMAR hatchery (France)

**Software and algorithms**		

STAR Mapper	https://github.com/alexdobin/STAR	
FeatureCounts	https://subread.sourceforge.net/featureCounts.html	
edgeR	https://bioconductor.org/packages/release/bioc/html/edgeR.html	
RUVseq	https://bioconductor.org/packages/release/bioc/html/RUVSeq.html	
clusterProfiler	https://bioconductor.org/packages/release/bioc/html/clusterProfiler.html	
Microbiomeanalyst	https://www.microbiomeanalyst.ca	

### Experimental model and study participant details

Adult individuals of Manila clam (*R*. *philippinarum*), with an average length of 26.01 ± 1.78 mm and an average weight of 3.75 ± 0.81 g, were purchased from SATMAR (France) in February 2022 and brought to the marine station of the University of Padua, located in Chioggia where all experiments were carried out. It was not possible to sex experimental animals used in the experiment. Experimental activities were carried out in 25L tanks with 30 clams per tank, ensuring at least 0.5 liters for each clam, and were filled with natural sea water from the Venice lagoon. For the entire duration of the experimental activities (Figure S1), about 50% of the water was replaced every two days, maintaining an average salinity of 30 PSU. Each tank was equipped with a pump, air stones, an iButton temperature logger (Maxim Integrated, USA, model: DS1921H-F5), and a 100 W heater connected to a precision thermoregulator (MPT91 Mect, Turin Italy) programmed to maintain the desired temperatures (i.e. either constant or fluctuating throughout the day). Clams were fed daily with the commercial mixture of microalgae New Coral Fito Concentrate (A.G.P., Italy), composed of *Isochrysis* (T-Iso) (33.3%), *Nannochloropsis* (31%), *Tetraselmis* (18%) and *Phaeodactylum* (18%), at a final concentration of ∼40 ∗ 10^6^ cell L^−1^.

### Method details

#### Experimental design

A 2x2 full factorial design was performed with “Priming” (levels: priming “P” and naÏve “N”) and “Marine HW” (levels: heatwave “H” and control “C”) as fixed factors. The experimental design is depicted in Figure S1.

Acclimation in experimental tanks lasted 15 days. During this period water temperature was gradually increased from 18 to 25°C (at the rate of 1°C day^-1^, until the target temperature was reached). Following acclimation, animals were tagged, weighted, and measured, then randomly divided into two groups: primed (P) and naÏve (N, i.e. non-primed, as defined by Hackerott et al.^31^). The temperature of the primed group was increased to 30°C (at the rate of 2.5°C day^-1^) and then kept constant at 30°C for 7 days, while the control group was maintained at 25°C, which represents the average summer water temperature recorded in the Venice lagoon. We chose a priming temperature of 30°C because it represents a sublethal stressful condition, as demonstrated previously,^26^ and a duration of 7 days to test the effectiveness of a long priming treatment on eliciting long-lasting memory effects. Subsequently, a resting period of 15 days (chosen because it represents the average gap between consecutive heatwaves in the Mediterranean Sea) at 25°C was carried for both groups to allow recovery from the priming conditions. Then each of the two treatments was randomly split in two groups: one was triggered with a simulated MHW (see below for additional details) while the other group was kept at 25°C as control. This experimental design resulted in four different conditions in total, namely PH: primed triggered with MHW; PC: primed non-triggered with MHW; NH: naÏve triggered with MHW; NC: naÏve non-triggered with MHW. Each of these four conditions was tested in duplicate, in 25 L aquaria with 30 clams per tank.

The simulated MHW lasted for 7 days, and water temperatures gradually oscillated each day from 31 to 35°C (Figure S1B). These oscillations mimicked a MHW recorded in the Venice lagoon in 2015 (https://www.marineheatwaves.org/tracker.html), except for the fact that temperatures were shifted upward +1°C to account for near future predictions of increase in water temperature due to CC.^66^ Daily thermal oscillations were obtained via programming the precision thermoregulators (MPT91 Mect, Turin Italy) equipped in each tank. Groups that were not exposed to the MHW were kept at 25°C. At the end of the MHW, temperatures were brought back to 25°C and kept constant for additional 15 days.

Animal survival was monitored daily throughout the duration of the experiment and for the 15 days following the end of the simulated MHW. The total duration of the experiment was 46 days.

#### Sample analysis

##### Behavioral tests

To assess the behavioral response of clams following the exposure to priming and HW, tests were conducted on a random subset of 25 clams for each group. The test was carried out twice: at the end of the priming treatment (i.e. day 8, Figure S1A) and at the end of the HW (i.e. day 32, Figure S1A). In accordance with Peruzza et al.*,*^26^ for each behavioral test an 80L tank was prepared by adding four centimeters of sand at the bottom of the tank and topped with 10 centimeters of seawater and equipped with a small pump and aerator. A Logitech C920 PRO HD camera (Logitech, Switzerland) was positioned above each tank, and once the clams were placed on the sand, they were filmed for 2 hours in agreement with.^67^ During the first behavioral test, water temperature was maintained at 30°C for primed animals and at 25°C for naÏve animals. The second behavioral test, performed at the end of the MHW, was carried at the respective temperature of each experimental group to avoid that abrupt changes in temperature could interfere with animal’s behavior (i.e. water temperature was 25°C for groups PC and NC while it was 31°C for PH and NH groups).

Behavioral videos were double-blind analyzed, recording for each clam the time it was placed on the sand, the time of the first recorded movement, and the time the clam had successfully completed burrowing in the sand, in accordance with Peruzza et al*.*^26^ This allowed to calculate the percentage of clams that successfully completed the burrowing and the time taken for this process.

##### Physiological and biochemical assays

The physiological status of clams was assessed by looking at their condition index. This index was calculated at the end of the experiment (i.e. day 46, Figure S1) on all surviving clams from the four groups. With the help of a scalpel, the shell was separated from the soft body tissue, which was then blotted on paper and weighed on a precision scale (Sartorius, Fisher Scientific). Similarly, the shell was blotted on paper and weighed. The condition index, defined as the weight of the soft tissue over the weight of the shell, is a measure of the general well-being of the clam.

At the end of the experiment, the mantle of 40 clams, 10 per group, was cut into two pieces, snap frozen on dry ice and stored at -80°C. These samples were later used to evaluate the extent of lipid degradation and the activity of two enzymes produced in response to oxidative stress: glutathione peroxidase (GPx) and superoxide dismutase (SOD). The assays used for these analyses are based on spectrophotometric techniques: the absorbance of a reaction product or of an intermediate is measured to derive its concentration through the construction of a calibration curve with known concentration standards. All samples were run in duplicate using transparent 96-well plates (SARSTEDT, model 82.1582.001) and a microplate spectrophotometer (Multiskan Go1510, Thermo Fisher).

First, tissue homogenate was prepared from the mantle tissue. About 10 mg of sample was immersed in 300 μL of homogenization buffer (containing NaCl 150 mM, EGTA, 1 mM EDTA 1 mM pH 8, TrisHCl 10 mM pH 7.4, TritonX100 0.2%, Phosphatase Inhibition Cocktail, Nonidet 5%, PIC 1x, and H_2_O) with metal beads using the TissueLyser II (QIAGEN, Germany). The samples were then centrifuged at 4°C for 20 minutes at 10000 rcf and the supernatant was taken. Total protein content was determined via Bradford method (Bradford Reagent, B6916, Sigma). The remaining homogenate was aliquoted and stored at -80°C for subsequent processing.

To measure SOD activity, the Dismutase Activity Assay Kit (CS0009, Sigma) was used following the manufacturer’s instructions.

The glutathione peroxidase activity was determined indirectly by assessing the amount of NADPH consumed in the reducing reaction of oxidized glutathione (GSSG) to reduced glutathione (GSH). GSH is the substrate for glutathione peroxidase (GPx) and gets oxidized to GSSG. Six standards of known NADPH concentrations were obtained by diluting 1 mM NAPDH with a diluting solution composed of PBS 62.5 mM, EDTA 6.25 mM, H2O. GPx 2 U/ml (G6137-100UN, Sigma Aldrich) was used as positive control and the diluting solution was used as reagent control. All samples, standards, and controls were run in duplicate. 25 μL of each sample were put in the sample wells and 80 μL of the assay solution containing all the necessary reagents to recreate the GSSG reduction and GSH oxidation reaction (GSH 130 mM, GR 2.5 U/ml, NADPH 40 mM (481973-25MG, Sigma Aldrich) suspended in the diluting solution) were added to the samples and controls wells. The plate was shaken for 15 minutes at room temperature, and to ensure an excess of NADPH in the samples, if the optical density in the wells was less than 1.0, 1 μL of 40 nM NADPH was added. The GSH oxidation reaction by GPx was initiated by adding 20 μL of 1.875 mM cumene hydroperoxide (247502-5G, Sigma Aldrich). Finally, absorbance at 340 nm was read for 10 minutes to determine how much NADPH was consumed in that time interval. A calibration curve was obtained by the standard absorbances to get the concentration of NADPH in the samples and to derive GPx activity.

Lipid peroxidation was estimated by measuring the concentration of the final product of a polyunsaturated lipid peroxidation, malondialdehyde (MDA), in accordance with the protocol of a commercial kit (MAK085, Sigma Aldrich).

##### RNAseq analyses

At the end of the experiment, from the same animals from which the mantle was taken for lipid and enzymatic assays, the digestive gland was collected and stored at -20°C in RNAlater® (ThermoFisher, USA). RNA was extracted using the RNeasy Mini Kit (Qiagen, Germany), quantified with Qubit Fluorometer (Invitrogen, USA) and qualitatively assessed with the Bioanalyzer (Agilent). RNA samples with concentration >20 ng/μl and RIN > 8 were sent to CRIBI (University of Padua, Italy) for the preparation of 3’ Tag Lexogen (Lexogen GmbH, Austria) library and sequencing. Sequencing was performed on Illumina Novaseq 6000 platform with 75 SE sequencing. The sequences obtained are available in NCBI Sequence Read Archive (BioProject ID: PRJNA1132140).

The quality of the raw reads was assessed using FastQC/v0.11.9 (https://www.bioinformatics.babraham.ac.uk/projects/fastqc/) and BBduk of the suite BBTools was used for trimming. STAR/v2.7.11a^68^ was employed to map the trimmed reads onto the Manila clam genome from Xu et al.^69^ with the option “--outFilterMismatchNoverLmax=0.1”. Gene counts were obtained using the program featureCounts/v2.0.0^70^ by counting multimapping reads with options “-M” and “--fractional” and the count table was analyzed with R/v4.0^71^ to determine differentially expressed genes between four pairs: NH vs NC, PH vs PC, PH vs NH, PC vs NC. Before conducting the pairwise differential expression analysis, transcriptome data was filtered to remove genes with low expression values that could contribute to background noise in accordance with Bernardini et al.,^72^ data were then normalized using the 'RUVs' function of the RUVSeq/v1.36 package,^73^ selecting respectively 1, 2, 6 and 3 factors of unwanted variation for each pairwise comparison.

Pairwise differential expression analysis was performed using the R package edgeR/v4.0^74^ running a likelihood ratio test (LRT) to identify differentially expressed genes. From the differential expression result file containing all genes, only those with an adjusted FDR < 0.05 and a log2FC threshold > |0.58| were deemed significant.

To determine if there were overrepresented categories among the differentially expressed genes, a Gene-Set Enrichment Analysis (GSEA) was performed to test for enriched functions using the software clusterProfiler/v4.6.2^75^ and the databases: Gene Ontology, KEGG, and Reactome. The functional databases were downloaded from gProfiler’s webpage (https://biit.cs.ut.ee/gprofiler/gost) using human as reference species. Identity between human genes and Manila clam genes was assessed by running Blastx search as specified in Peruzza et al*.*^26^ For these analyses, the FDR threshold was set at 0.2.

##### Clam-associated microbiota

Microbiota analyses were carried on the same RNA used for transcriptomic analysis. Preparation for the biological material followed steps described in Peruzza et al*.*^26^ The 16S amplicon sequencing were performed at Biomarker Technologies facility (BMK GmbH, Germany) targeting V3-V4 region of 16S gene, all samples were then sequenced on Illumina Novaseq 6000 with 250 pair-end approach. The sequences obtained are available in NCBI Sequence Read Archive (BioProject ID: PRJNA1132140).

Sequences were analyzed using the pipeline of QIIME2,^76^ which constated in filtering raw reads based on quality score, dereplication, merging and chimera removal by using dada2/v1.14.1,^77^ as reported in detail in Peruzza et al*.*^26^ ASVs were taxonomically classified up to genus level using the SILVA 16S Database (NR98 version 138.1). The ASV table was then imported in microbiomeanalyst (https://www.microbiomeanalyst.ca) where it was further processed. ASV table was filtered to remove low count ASV with less than 4 counts in 10% of the samples. ASV table was not rarefied, in accordance with Willis.^78^ Alpha and beta diversity metrices were calculated using default values. Pairwise comparisons were performed using the “Multiple Linear Regression with Covariate Adjustment” tool and ASV with FDR < 0.05 were deemed significant.

### Quantification and statistical analysis

Differences in survival between treatments were assessed and plotted using the survminer/v0.4 library in R.

For each quantified parameter, normality and homogeneity of variances were assessed using the “shapiro.test” and “var.test” functions of base R. When normality and/or homogeneity of variances were not met, an attempt to normalise them was performed using the bestNormalize/v1.8 library.

Behavioural analyses in relation to the proportion of clams that completed burying were analysed and plotted using the “ggbarstats” function from the ggstatsplot/v0.8^79^ library. Behavioural analyses relating the burying speed were analysed and plotted either using the “ggbetweenstats” function, or by using a custom 2-Way ANOVA code in R followed by plots with ggplot2.^80^

Physiological and biochemical data were analyzed using a 2-way ANOVA: when normality and homogeneity of variances were granted, a classical 2-way ANOVA was performed with post-hoc tests in R; if data were not normally distributed, pairwise comparisons using Wilcoxon rank sum exact test were performed. An adjusted p-value <0.05 was deemed significant. Detailed results of the 2-way ANOVA analyses are reported in Data S1 (see Supplemental Item). Post-hoc pairwise comparisons results are integrated within each plot by means of the compact letter display method where groups sharing the same letter are not significantly different from each other, while groups with different letters have statistically significant differences.

## Supplemental information

Document S1. Figure S1 and Data S1

Table S1. Transcriptomic analyses

Table S2. 16S metabarcoding analyses
