# Supplementary material for: Thermal priming mitigates the effects of lethal marine heatwaves on the Manila clam Ruditapes philippinarum
Source: iScience. 2025 Jul 16;28(8):113108. doi: 10.1016/j.isci.2025.113108 (PMC12314336; doi:10.1016/j.isci.2025.113108)
Supplement: Document S1. Figure S1 and Data S1 [file mmc1.pdf]

iScience, Volume *28*

Supplemental information

Thermal priming mitigates the effects of lethal marine heatwaves on the Manila clam *Ruditapes philippinarum*

Carmen Federica Tucci, Massimo Milan, Giulia Dalla Rovere, Ilaria Bernardini, Serena Ferraresso, Rafaella Franch, Massimiliano Babbucci, Giovanna Monticelli, Mattia Panin, Tomaso Patarnello, Luca Bargelloni, and Luca Peruzza

Supplemental figures:

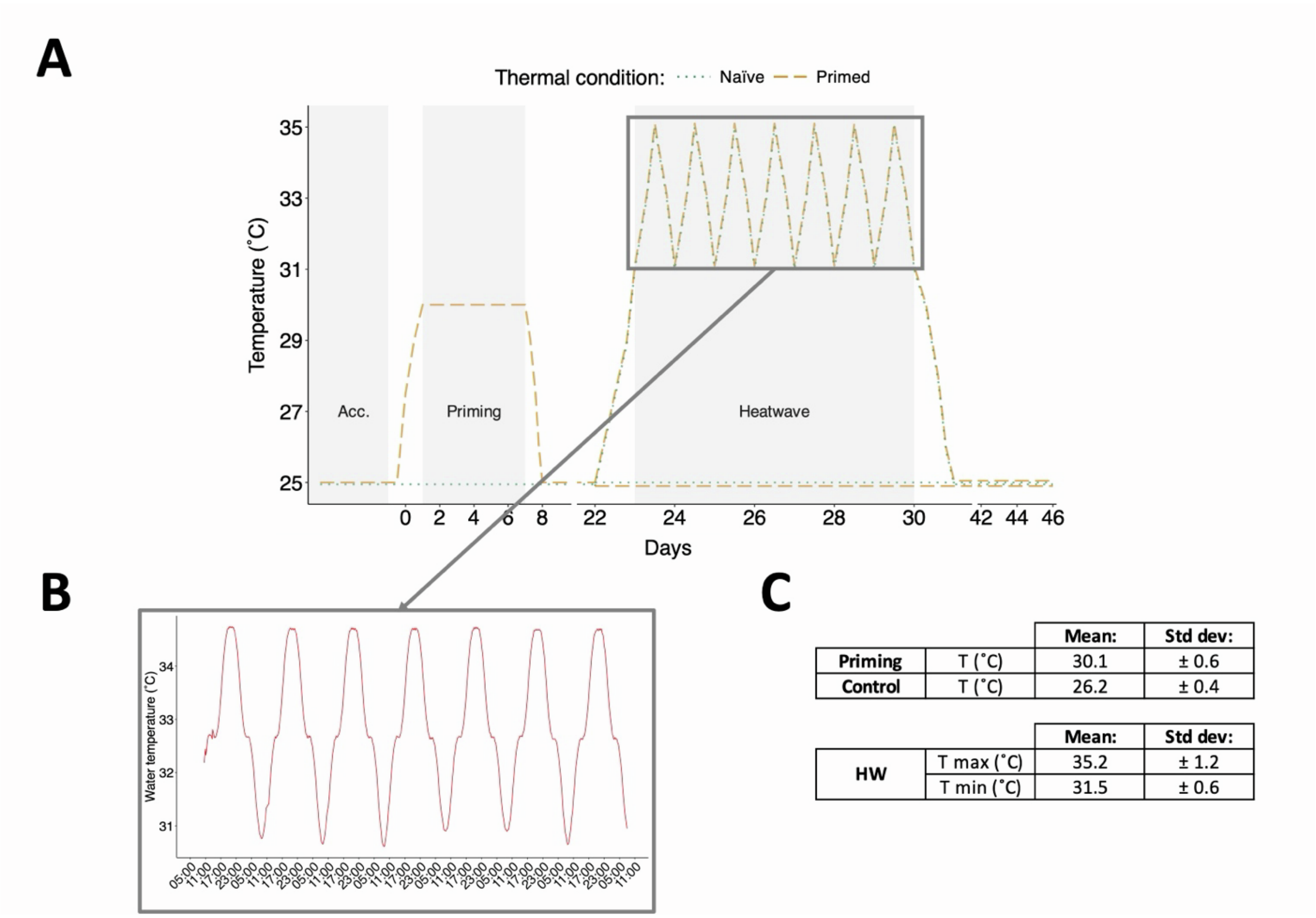

		Mean:	Std dev:
**Priming**	T (°C)	30.1	± 0.6
**Control**	T (°C)	26.2	± 0.4

		Mean:	Std dev:
**HW**	T max (°C)	35.2	± 1.2
	T min (°C)	31.5	± 0.6

Supplementary Figure 1: Experimental scheme. A) Experimental design. Shaded areas indicate different stages of the experiment: “Acc” indicates acclimation period before the beginning of the experiment; “Priming” indicates the duration of the priming treatment; “Heatwave” indicates the duration of the MHW treatment. B) Hourly thermal profile implied during the MHW conditions. This thermal profile was designed to mimic a MHW recorded in the Venice lagoon in 2015. Temperatures were shifted upward by +1 ˚C to account for predicted average increase in water temperatures due to CC [1]. C) Table with mean water temperature ± SD measured in different stages of the experiment across all tanks.

Data S1:

Data S1: Two-way ANOVA analyses results.

Condition Index:

Testing Normality Assumption with Shapiro-Wilk

```
        Shapiro-Wilk normality test

data:  MyAOV_residuals
W = 0.9849, p-value = 0.22
```

Testing Homogeneity of Variance with Brown-Forsythe:

```
Brown-Forsythe Test for Homogeneity of Variance using median
      Df F value  Pr(>F)
group   3  2.9609 0.03533 *
      112
---
Signif. codes:  0 ‘***’ 0.001 ‘**’ 0.01 ‘*’ 0.05 ‘.’ 0.1 ‘ ’ 1
```

Data were normally distributed. An ANOVA was performed:

ANOVA TABLE:

term	sumsq	meansq	df	statistic	p.value	etasq	partial.etasq	omegasq	partial.omegasq	epsilonsq	cohens.f	power
Challenge	167.528	167.528	1	10.758	0.001	0.062	0.088	0.056	0.078	0.057	0.310	0.907
Treatment	717.493	717.493	1	46.075	< .001	0.268	0.291	0.260	0.280	0.262	0.641	1.000

Challenge:Treatment | 52.961 | 52.961 | 1 | 3.401 | 0.068 | 0.020 | 0.029 | 0.014 | 0.020 | 0.014 | 0.174 | 0.454

Residuals | 1744.094 | 15.572 | 112 | | | | | | | | |

Posthoc multiple comparisons of means: Scheffe Test

95% family-wise confidence level

$Challenge

diff lwr.ci upr.ci pval

NoChallenge-Challenge 2.578499 0.4981973 4.6588 0.0081 **

$Treatment

diff lwr.ci upr.ci pval

Priming-Control -4.971774 -7.052076 -2.891473 2e-08 ***

Signif. codes: 0 '***' 0.001 '**' 0.01 '*' 0.05 '.' 0.1 ' ' 1

Behaviour after MHW - bury speed:

Data were not normally distributed. A non-parametric ANOVA was used with Kruskal-Wallis rank sum test and Wilcoxon rank sum exact test:

Joining Challenge and Treatment into the variable 'Combined'

Kruskal-Wallis rank sum test

data: Elapsed_minutes_transf by Combined

Kruskal-Wallis chi-squared = 9.74, df = 3, p-value = 0.02091

Pairwise comparisons using Wilcoxon rank sum exact test:

data: dataframe[, depvar] and dataframe[, "Combined"]

	Control_Naïve	Control_Primed	Heatwave_Naïve
Control_Primed	0.478	-	-
Heatwave_Naïve	1.000	1.000	-
Heatwave_Primed	0.014	1.000	1.000

P value adjustment method: bonferroni

GPx activity:

Data were not normally distributed. A non-parametric ANOVA was used with Kruskal-Wallis rank sum test and Wilcoxon rank sum exact test:

Pairwise comparisons using Wilcoxon rank sum exact test

data: df$GPx_norm and df$Combination

	Heatwave_Naïve	Heatwave_Priming	Control_Naïve
Heatwave_Priming	0.0047	-	-
Control_Naïve	1.0000	0.0152	-
Control_Priming	0.0058	0.6717	0.0148

P value adjustment method: bonferroni

SOD activity:

Shapiro-Wilk normality test

data: MyAOV_residuals

W = 0.97842, p-value = 0.631

Brown-Forsythe Test for Homogeneity of Variance using median

```
      Df F value Pr(>F)
group  3  2.1026  0.117
      36
```

Data were normally distributed. An ANOVA was performed:

ANOVA TABLE:

term	sumsq	meansq	df	statistic	p.value	etasq	partial.etasq	omegasq	partial.omegasq	epsilonsq	cohens.f	power
Challenge	2.056	2.056	1	3.071	0.088	0.039	0.079	0.026	0.049	0.026	0.292	0.418
Treatment	24.064	24.064	1	35.947	< .001	0.451	0.500	0.433	0.466	0.438	0.999	1.000
Challenge:Treatment	3.167	3.167	1	4.730	0.036	0.059	0.116	0.046	0.085	0.047	0.362	0.585
Residuals	24.100	0.669	36									

Posthoc multiple comparisons of means : Tukey HSD

   95% family-wise confidence level

$Treatment

```
                    diff   lwr.ci   upr.ci    pval
Priming-Control 1.558101 1.030718 2.085484 7.1e-07 ***
```

```
$`Challenge:Treatment`
                                   diff      lwr.ci   upr.ci    pval
Control:Naïve-Heatwave:Naïve      1.08187603  0.03662669 2.127125  0.0402 *
Heatwave:Priming-Heatwave:Naïve       2.04996123  1.12308684 2.976836 4.6e-06 ***
Control:Priming-Heatwave:Naïve     1.98817927  0.97570455 3.000654 3.6e-05 ***
Heatwave:Priming-Control:Naïve     0.96808521 -0.02211182 1.958282  0.0573 .
Control:Priming-Control:Naïve  0.90630324 -0.16444372 1.977050  0.1219
Control:Priming-Heatwave:Priming    -0.06178197 -1.01731796 0.893754  0.9981

---
Signif. codes:  0 '***' 0.001 '**' 0.01 '*' 0.05 '.' 0.1 ' ' 1
```

Lipid Peroxidation activity:

Brown-Forsythe Test for Homogeneity of Variance using median

```
      Df F value  Pr(>F)
group  3  2.3025 0.09051 .
      43
---
Signif. codes:  0 ‘***’ 0.001 ‘**’ 0.01 ‘*’ 0.05 ‘.’ 0.1 ‘ ’ 1
```

Shapiro-Wilk normality test

```
data:  MyAOV_residuals
W = 0.9658, p-value = 0.1823
```

Data were normally distributed. An ANOVA was performed:

ANOVA TABLE:

term	sumsq	meansq	df	statistic	p.value	etasq	partial.etasq	omegasq	partial.omegasq	epsilonsq	cohens.f	power
Challenge	0.109	0.109	1	1.967	0.168	0.034	0.044	0.017	0.020	0.017	0.214	0.289
Treatment	0.003	0.003	1	0.048	0.828	0.001	0.001	-0.016	-0.021	-0.017	0.033	0.055
Challenge:Treatment	0.682	0.682	1	12.336	0.001	0.215	0.223	0.194	0.194	0.198	0.536	0.940
Residuals	2.377	0.055	43									

Posthoc multiple comparisons of means : Tukey HSD

95% family-wise confidence level

$\`Challenge:Treatment\`

	diff	lwr.ci	upr.ci	pval	
Control:Naïve-Heatwave:Naïve	-0.177927375	-0.465555263	0.10970051	0.3606	
Heatwave:Priming-Heatwave:Naïve	-0.187337147	-0.413171252	0.03849696	0.1349	
Control:Priming-Heatwave:Naïve	0.145916285	-0.119027074	0.41085964	0.4630	
Heatwave:Priming-Control:Naïve	-0.009409772	-0.294163362	0.27534382	0.9997	
Control:Priming-Control:Naïve	0.323843660	0.007175778	0.64051154	0.0433	*
Control:Priming-Heatwave:Priming	0.333253432	0.071433287	0.59507358	0.0077	**

Signif. codes: 0 '***' 0.001 '**' 0.01 '*' 0.05 '.' 0.1 ' ' 1

References:

1. Ferrarin, C., Bajo, M., Bellafiore, D., Cucco, A., De Pascalis, F., Ghezzo, M., and Umgiesser, G. (2014). Toward homogenization of Mediterranean lagoons and their loss of hydrodiversity. Geophys. Res. Lett. *41*, 5935-5941. 10.1002/2014gl060843.
